# Expression of cold-inducible RNA-binding protein in mouse spinal cord injury model

**DOI:** 10.1371/journal.pone.0311803

**Published:** 2025-03-04

**Authors:** Xinhui Zhang, Yi Zhao, Jing Guo, Jingkun Chen, Xue Gao, Wentao Pan, Hengli Li, Shutong Yao, Yueying Zhang

**Affiliations:** 1 The First Affiliated Hospital of Shandong First Medical University, Jinan, Shandong, China; 2 Department of Pathophysiology, School of Clinical and Basic Medicine, Shandong First Medical University & Shandong Academy of Medical Sciences, Jinan, Shandong, China; 3 Intensive Care Medicine Department, The Affiliated Taian City Central Hospital of Qingdao University, Taian, Shandong, China; The Second Affiliated Hospital of Guangzhou Medical University, CHINA

## Abstract

**Objective:**

To investigate the changes of **Cold-Inducible RNA-Binding Protein** (CIRBP) expression in mouse spinal cord injury model.

**Methods:**

Seventy-five female C57BL/6 mice were randomly divided into five groups, 15 mice per group. According to different degrees of spinal cord injury, they were divided into Mild spinal cord injury, Moderate spinal cord injury, Severe spinal cord injury, Spinal cord amputation group, and Sham surgery group, all constructed with spinal cord percussion. All groups were dissected 1, 3, 5, 14, and 21 days after modeling. HE staining was used to observe the pathological changes in the spinal cord, The Basso mouse scale (BMS) was used for motor function scoring, and immunofluorescence was used to detect the expression of NeuN, IBA-1, and CIRBP in spinal cord tissues.

**Results:**

HE results showed that inflammation was more pronounced in moderate, severe, and amputation injuries compared to the Sham surgery group. Moderate injury group and Severe injury group inflammation increased consistently over time. The severe injury group had severe tissue structure destruction and increased astrocytes significantly. Combined with the mouse BMS motor function score, the mouse severe injury group model was more stable. Compared with the Sham surgery group, there was a significant decrease in NeuN over time (P < 0.01) and a significant increase in IBA-1 and CIRBP (P < 0.01) in the severe injury group. Moreover, IBA-1 has significant co-localization with CIRBP.

**Conclusion:**

CIRBP expression is significantly elevated in a mouse spinal cord injury model, Which may be related to the proliferation of microglia during spinal cord injury.

## 1. Introduction

Spinal Cord Injury (SCI) is a disease that occurs mainly in adolescents. In recent years, the age of onset of the disease in Asia is mainly between 30-40 years old [1]. SCI is categorized into primary and secondary injuries. Secondary injuries are secondary lesions that occur on top of the primary injury. Primary injury is the most primitive injury due to direct mechanical [1,2]. Primary injuries result in axonal rupture necrosis of cells within the spinal cord and are categorized into several conditions, such as spinal cord concussion (SCC) [3], spinal cord contusion [4], hemorrhage, spinal cord rupture [5], spinal cord compression, and cauda equina injury, which often result in permanent and irreversible damage [6]. There are no effective medications to intervene. Disruption of the blood-brain-spinal cord barrier, prolonged hematoma, and hemorrhagic necrosis, all of which create an inflammatory environment and attract large numbers of inflammatory cells, such as neutrophils, microglia, and T cells [2,7]. Inflammatory cells secrete large amounts of inflammatory factors such as tumor necrosis factor-α (TNF-α), interleukin (IL)-1α, IL-1β, and IL-6, resulting in a more severe inflammatory response [8,9].

Cold-induced RNA binding protein (CIRBP) belongs to the cold shock protein family, which consists of an amino-terminal shared sequence RNA-binding structural domain and a carboxy-terminal glycine-rich structural domain and acts as an RNA chaperone to facilitate translation. In recent years, reports have shown that CIRBP is a damage-associated molecular pattern (DAMP) that exhibits pro-inflammatory properties in several diseases (e.g., liver injury, kidney injury, and colitis) and can cause various degrees of tissue and organ damage [6]. Clinically, therapeutic hypothermia (32°C ~ 34°C) is a means to alleviate the neurological deficits in children with hypoxic-ischemic encephalopathy and adult acute brain injury, and the synthesis of cirbp protein reaches its peak at mild to moderate hypothermia (32°C ~ 34°C), so cirbp plays a crucial role in it [10]. Currently, cirbp is widely studied in neurological diseases. However, the study of cirbp in SCI has not yet been reported, so it is significant to study the expression and application of cirbp in SCI, which will lay the foundation for future clinical application. In this study, we constructed a mouse model of spinal cord injury. We explored its possible role in spinal cord injury through immunofluorescence expression to provide an experimental basis for the mechanism of spinal cord injury and the search for prevention and treatment targets.

## 2. Materials and methods

### 2.1. Ethics statements

All experimental protocols and animal handling procedures are approved and supervised by the Laboratory Animal Management Committee of Shandong First Medical University & Shandong Academy of Medical Sciences (2021 No.192) and animal experimentation approval number is: W202311160320. The experiment is in line with the Measures for the Implementation of the Regulations on the Administration of Laboratory Animals in Shandong Province (No.41; No.311). Animal welfare complies with the Technical Specification for Building Experimental Animal Facilities issued by the Ministry of Housing and Urban-Rural Development of the People’s Republic of China/General Administration of Quality Supervision, Inspection and Quarantine of the People’s Republic of China (GB 50447-2008). Animals were anesthetized to death by intraperitoneal injection of an overdose of 1% sodium pentobarbital prior to sampling.

### 2.2. Construction of mouse spinal cord injury model

Adult female C57/BL Mouse (20–25 g, n = 75) were deeply anesthetized by an intraperitoneal (i.p.) injection of 1% pentobarbital sodium (50 mg/kg). A laminectomy was performed to expose the T10 spinal segment. The spine was immobilized with a spinal immobilizer, and model preparation was performed using a spinal cord striker (68100, RWD). The Sham group was only exposed without strikes. The modeling strike depths and times for the different model groups are shown in the following (Table 1). After hemostasis was achieved, the overlying musculature and skin were sequentially sutured. The animals received extensive post-operative care, including intramuscular injection of penicillin (50,000 U/kg per day) for three days and manual emiction two times daily until automatic micturition function was re-established.

**Table 1:** Mouse spinal cord injury strike parame.

	Sham	Mild injury	Moderate injury	Moderate injury	Complete injury
Injury Depth (mm)	–	0.3–0.4	0.5–0.6	1.0–1.2	2.2
Residence time (s)	–	0.4	0.4	0.4	0.4
Injury Speed (m/s)	–	1~2	1~2	1~2	1~2

### 2.3. Behavioral test

Mice were subjected to BMS (basso mouse scale, BMS) testing at each period before surgery and after SCI. Briefly, double-masked scoring was used, and two persons not involved in the experiments of this subject observed the mice’s movements to assess the frequency of hindlimb movements, joint range of motion, weight-bearing, and coordination.

### 2.4. Tissue processing

All mice were deeply anesthetized with 1% sodium pentobarbital (50 mg/kg, i.p.). Then, mice were first perfused intracardially with saline containing heparin and 0.002% NaNO2 and then with fixative in 0.1 M phosphate buffer (PB, pH 7.4) containing 4% paraformaldehyde. Tissue from the T10 spinal cord site was removed and post-fixed in fresh fixative for 48 h. Subsequently, the tissue was subjected to gradient dehydration, and the dehydration procedure was as follows: 40% ethanol overnight, 50% ethanol for four h, 60% ethanol for four h, 70% ethanol for three h, 80% ethanol for two h; 90% ethanol for two h; 95% ethanol I for 45 min; 95% ethanol II for 45 min; 100% ethanol I for 35 min; 100% ethanol II for 25 min; and xylene I for 25 min; Xylene II 20 min; paraffin I 30 min; paraffin II 30 min. After dehydration, the spinal cord was wrapped in paraffin wax, and paraffin sectioned at a thickness of 4 μm for subsequent experiments.

### 2.5. Hematoxylin-eosin staining

Paraffin sections were dewaxed gradient water, washed three times with PBS for five each time, and washed once with pure water for 5 min; placed in Harris hematoxylin for staining for 1 min, and washed in natural water for 1 min; 1% hydrochloric acid alcoholic differentiation solution was differentiated for 30 s, and washed with tap water for 5 min; sections were placed in eosin staining for 2 min, and were stained with 85% alcohol for 5 s, 90% alcohol for 5 s, 95% Alcohol I 1 min, 95% Alcohol II 1 min, anhydrous ethanol I 3 min, anhydrous ethanol II 3 min, xylene I 2 min, xylene II 2 min, and neutral gum sealing. The slides were visualized under a 3D pathology slide scanner (Hungary).

### 2.6. Nissl stain

Sections were dewaxed and hydrated, stained with 1% methylene blue staining solution (G1303, Solarbio) for 10 min, washed with distilled water, differentiated utilizing 70% alcohol for 2 min, and sequentially subjected to 80% alcohol for 2 min, 95% alcohol for 2 min, 100% alcohol I for 5 min, 100% alcohol II for 5 min, xylene I for 10 min, xylene II for 10 min, and sealing with neutral gum.

### 2.7. Immunofluorescence

Paraffin sections were baked for one h, deparaffinized in evident agent for three h, sequentially passed through the water preparation from high to low concentration of alcohol, and washed with PBS buffer three times for five each time; repaired in autoclave for 20 min using EDTA antigen repair solution, and washed with PBS buffer three times for 5 min each time; Incubate with 5% goat serum for 30 min at 37 °C; Antibodies were incubated for NeuN (Abcam, 104224) and CIRBP (Bioss, bs-7790R), IBA1 (Abcam, 5076) and CIRBP (Bioss, bs-7790R) for four °C overnight. The next day, rewarmed for one hour, PBS buffer was washed thrice for five each. Secondary antibodies were goat anti-mouse 488 (Affinity, S0018) and goat anti-rabbit 594 (Affinity, S0006), incubated at 37°C for one hour in a thermostat, and washed three times with PBS buffer for five each. Sections were sealed with a DAPI-containing sealer agent. Changes were observed under a 3D pathology slide scanner.

### 2.8. Tunel staining

Immunofluorescence of NeuN was performed as in step 2.7 above. The secondary antibody was incubated for one hour and washed thrice with PBS for 5 min each time. A pre-configured Tunel’s working solution (Beyotime, c1086) was added and incubated for one hour and washed three times with PBS for 5 min each time. Sections were sealed with a DAPI-containing sealer agent. Changes were observed under a 3D pathology slide scanner.

### 2.9. Statistical analysis

Mouse locomotor ability scores were double-blind scored, and raters complemented each other to analyze comparisons between different groups using the independent samples t-test, and econometric data were expressed as X̅ ± S; *p* < 0.05, the difference was statistically significant. All statistical graphs were plotted using GraphPad Prism 9.0 software.

## 3. Results

### 3.1. Constructing the T10 spinal cord injury model

**Fig 1:**
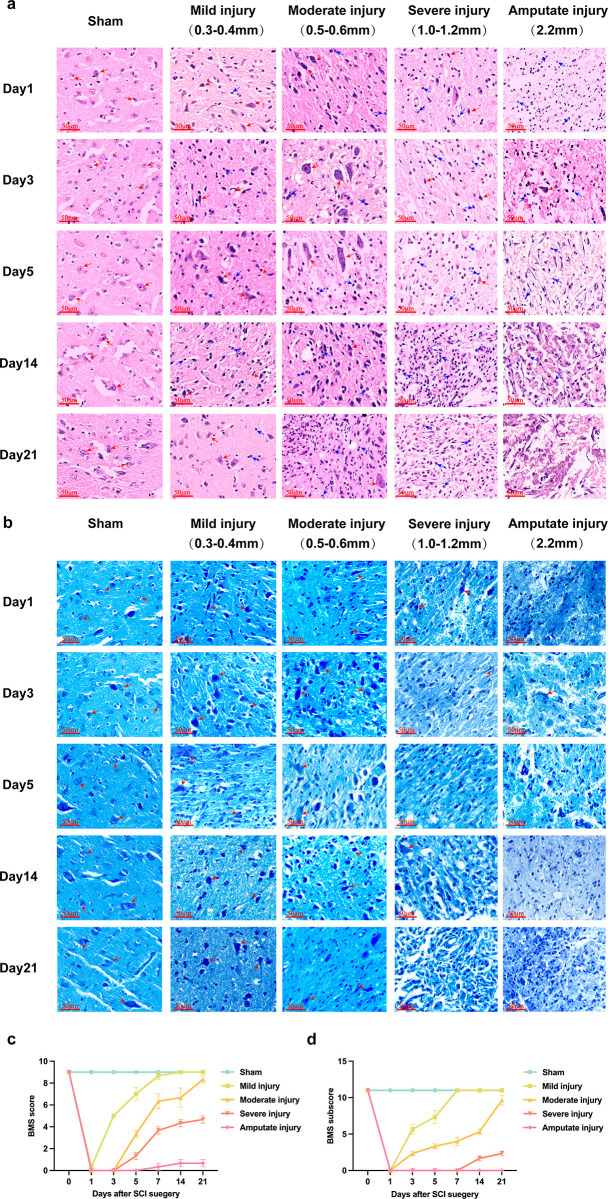
Constructing the T10 spinal cord injury model. a. HE results in Sham and different SCI groups (scale 50 μm), red arrows indicate neurons, blue arrows indicate inflammatory cells; b. Nissl stain results for Sham and different SCI groups (scale 50 μm), Red arrows indicate neurons; c. BMS score of locomotor performance in mice; d. BMS subscore of locomotor performance in mice.

The HE results showed that the Sham group had intact tissue structure and uniform cell distribution; compared with the Sham group, the number of neurons in the SCI group decreased and the tissue damage was aggravated as the depth of the blow increased (Fig 1a); Nissl stain results showed normal neurons with clear cytoplasm and homogeneous nuclei in the Sham group; the different SCI groups showed different loss of intact neuronal numbers, visible nuclear atrophy and disappearance of dorsal horn motor neuron cells (Fig 1b). Combined with mouse BMS scores (Fig 1c–d), severe injuries were more stable and compatible with the study.

### 3.2. Neuron apoptosis and increased expression of CIRBP

**Fig 2:**
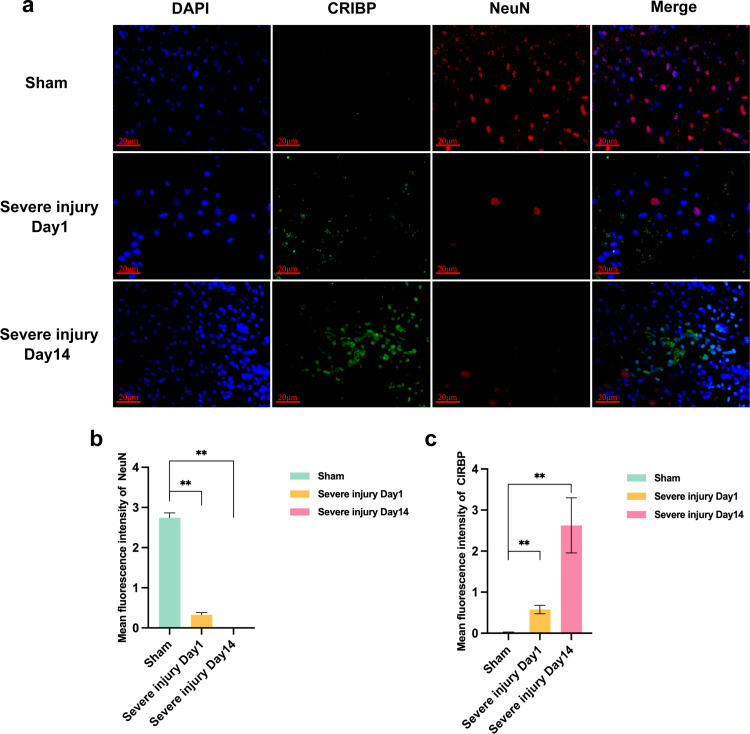
Expression of NeuN and CIRBP proteins in Sham and severely injured groups. a: Expression of NeuN and CIRBP, scale 20 μm. b: Mean fluorescence intensity of NeuN. Data are expressed as X̅ ± S; **P < 0.01; n = 3. c: Mean fluorescence intensity of CIRBP. Data are expressed as mean ± SEMs; **P < 0.01; n = 3.

Neurons play an uploading function in the spinal cord, so we examined the neuron-specific marker NeuN. Immunofluorescence results showed a significant decrease in NeuN expression and a gradual increase in CIRBP expression in the severely injured group compared to the Sham group on day 1 (Fig 2a–c).

### 3.3. Neuronal apoptosis

**Fig 3:**
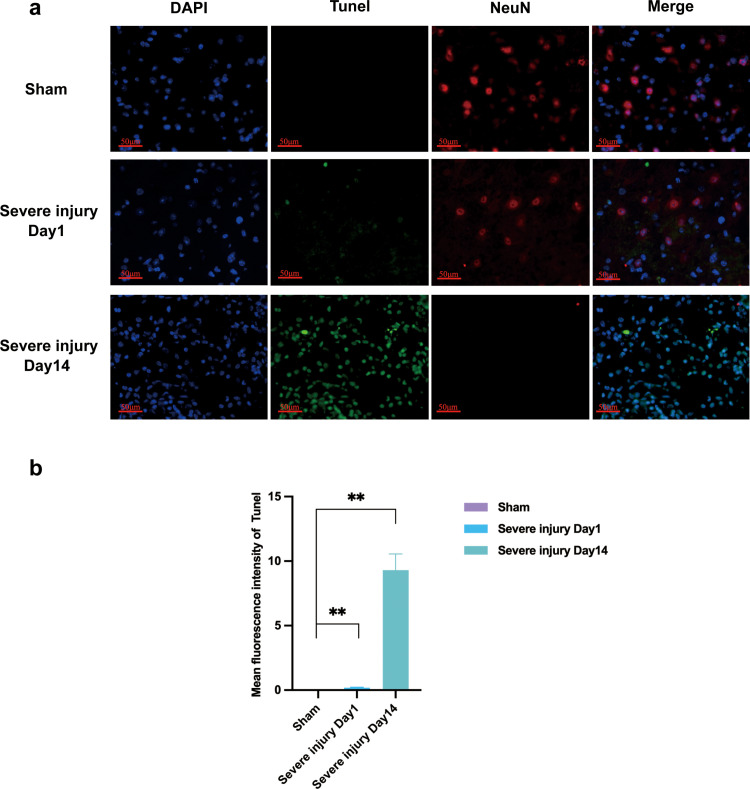
NeuN expression and neuronal apoptosis in Sham and severely injured groups. a: Expression of NeuN andTunel, scale 50 μm. b: Mean fluorescence intensity of Tunel. Data are expressed as X̅ ± S; **P < 0.01; n = 3.

Some studies have reported that neurons initiate an apoptotic program after spinal cord damage, and we detected the neuron-specific marker NeuN and apoptosis by immunofluorescence. Immunofluorescence results showed that neuronal apoptosis was significantly increased in the severely injured group on both the first day and the 14th day compared to the Sham group (Fig 3a,b).

### 3.4. Co-localization of IBA-1 and CIRBP expression

**Fig 4:**
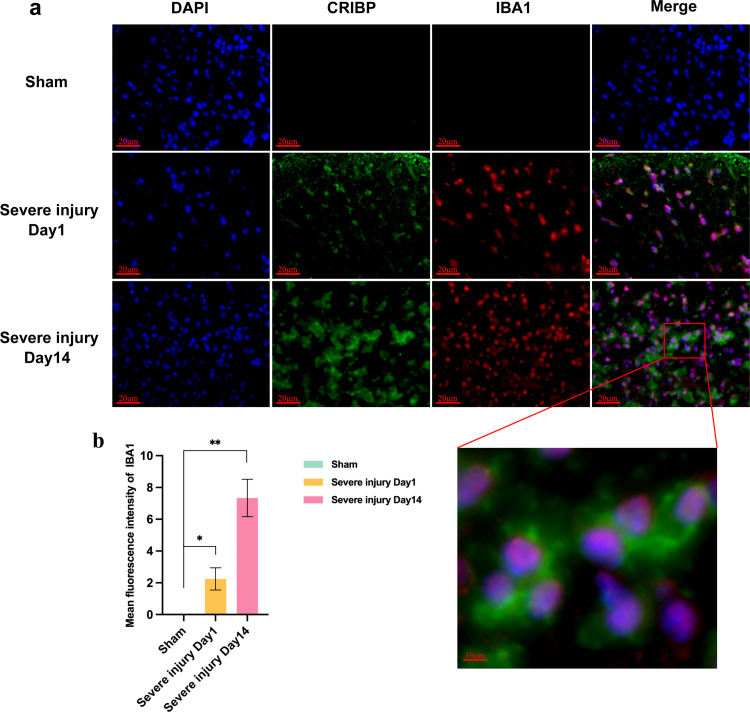
Expression of IBA1 and CIRBP proteins in Sham and severely injured groups. a: Expression of IBA1 and CIRBP, scale 20 μm. b. Mean fluorescence intensity of IBA1. Data are expressed as X̅ ± S;**P < 0.01; n = 3.

Studies have reported that CIRBP exhibits pro-inflammatory factor properties. We examined the expression of the microglia-specific marker IBA-1 by immunofluorescence. It was found that the expression of IBA-1 in the severe injury model gradually increased despite time. Meanwhile, we were surprised to find the co-localization of glial cell-specific markers IBA1 and CIRBP (Fig 4a–b).

## 4. Discussion

Spinal cord injury (SCI) is a complex neurological disorder. According to the study, the age-standardized rate of SCI is 13 cases per 100,000 people (11 to 16 cases) [11]. SCI is a typical result of multiple mechanisms after spinal cord injury and is categorized into primary and secondary injuries, with the latter mainly including acute, subacute, and chronic lesion phases [2,4,6]. Currently, the main clinical application of drugs for the treatment of spinal cord injury, but the effect is not good. The pathogenesis of spinal cord injury has not yet been elucidated; therefore, elucidating the mechanism of spinal cord injury and adopting effective preventive and curative measures has become a top priority in neurology. CIRBP, the first cold shock protein identified in mammals, has pro-inflammatory properties and induces tissue cell injury by promoting the formation and expression of pro-inflammatory cytokines, a damage-associated molecular pattern (DAMP) [12–15]. The study reported that CIRBP knockout mice were utilized to demonstrate that CIRBP deficiency significantly reduced levels of pro-inflammatory factors, myeloperoxidase, and apoptosis, attenuating the inflammatory response and leading to reduced injury [16]. We hypothesized that CIRBP may play an essential role in the acute phase of SCI.

Currently, there are many methods for SCI modeling, including contusion models, crush injury models, pulling and dislocation models, and transection models. Different modeling methods are chosen for different research purposes [15]. Allen successfully constructed the contusion model in 1991 using the Weight-dropping (WD) method, which is simple and easy to operate. However, the strike location will not be fixed, causing secondary injury. This study used the spinal cord strikers to screen the stable spinal cord injury model. The spinal cord can self-repair, and the severe injury model is more stable than other models, which meets the needs of subsequent experiments. HE and Nissl stain results showed that different injury strengths caused different spinal cord injury levels. A study demonstrates that a novel automated spinal cord injury contusion device for mice can accurately, reproducibly, and easily generate spinal cord injury contusion models. The system can accurately generate models of different levels of spinal cord injury, which is consistent with our aim of modeling different levels of injury [17]. Compared to the Sham group, the injury groups all showed different loss of intact neuronal numbers, visible nuclear atrophy, and loss of dorsal horn neuronal cells. Combined with the results of behavioral experiments, the model of the severe injury group was more stable compared with other model groups. In conclusion, the mouse spinal cord injury model was successfully established.

Studies have shown that elevated levels of ROS (reactive oxygen species) are a critical unfavorable factor for oxidative stress induction in cerebral ischemia-reperfusion injury. In PC12 cells, Cirbp expression decreases after H_2_O_2_ treatment to generate ROS. However, when Cirbp expression was elevated by endogenous or artificial means, apoptosis of neuronal cells induced by H_2_O_2_ could be significantly inhibited, suggesting a neuroprotective role for Cirbp [18,19]. In contrast to the beneficial effects in the cell, the release of Cirbp into the bloodstream is associated with a deleterious immune response. Both in vivo and in vitro experiments showed that Cirbp secreted by microglia after cerebral ischemia was associated with subsequent neuroinflammatory responses and neuronal damage due to Cirbp-mediated TNF-α expression [20]. The present study initially analyzed its expression changes by immunofluorescence technique. It was found that the expression level of CIRBP was significantly higher in the severely injured group compared to the Sham group and showed co-localization with microglia, which is similar to the results of previous studies [6,13,21–23]. Based on the experimental results, we hypothesized that the increased expression of CIRBP in the early stage of spinal cord injury might be associated with increased microglia, but the specific mechanism still needs further study. Exploring the role of CIRBP in spinal cord injury and the mechanism that triggers inflammation will not only bring hope to patients with spinal cord injury but also provide a reference for the treatment of other neurological disorders so that drugs targeting CIRBP can be developed for their benefit.

## 5. Conclusion

In summary, CIRBP expression was significantly elevated over time in spinal cord injury and significantly co-localized with IBA-1 expression, so it is speculated that CIRBP may be a key pro-inflammatory factor in the development of spinal cord injury; therefore, targeting CIRBP has the potential to be a clinical treatment for spinal cord injury.

## Supporting information

S1 File Analyze data. (ZIP)
