# Supplementary material for: Expression of cold-inducible RNA-binding protein in mouse spinal cord injury model
Source: PLoS One. 2025 Mar 4;20(3):e0311803. doi: 10.1371/journal.pone.0311803 (PMC11878929; doi:10.1371/journal.pone.0311803)
Supplement: S1 File — (ZIP) [file pone.0311803.s001.zip › Analyze Data/Illustration of HE staining and Nysted staining.docx]

**1.HE staining**


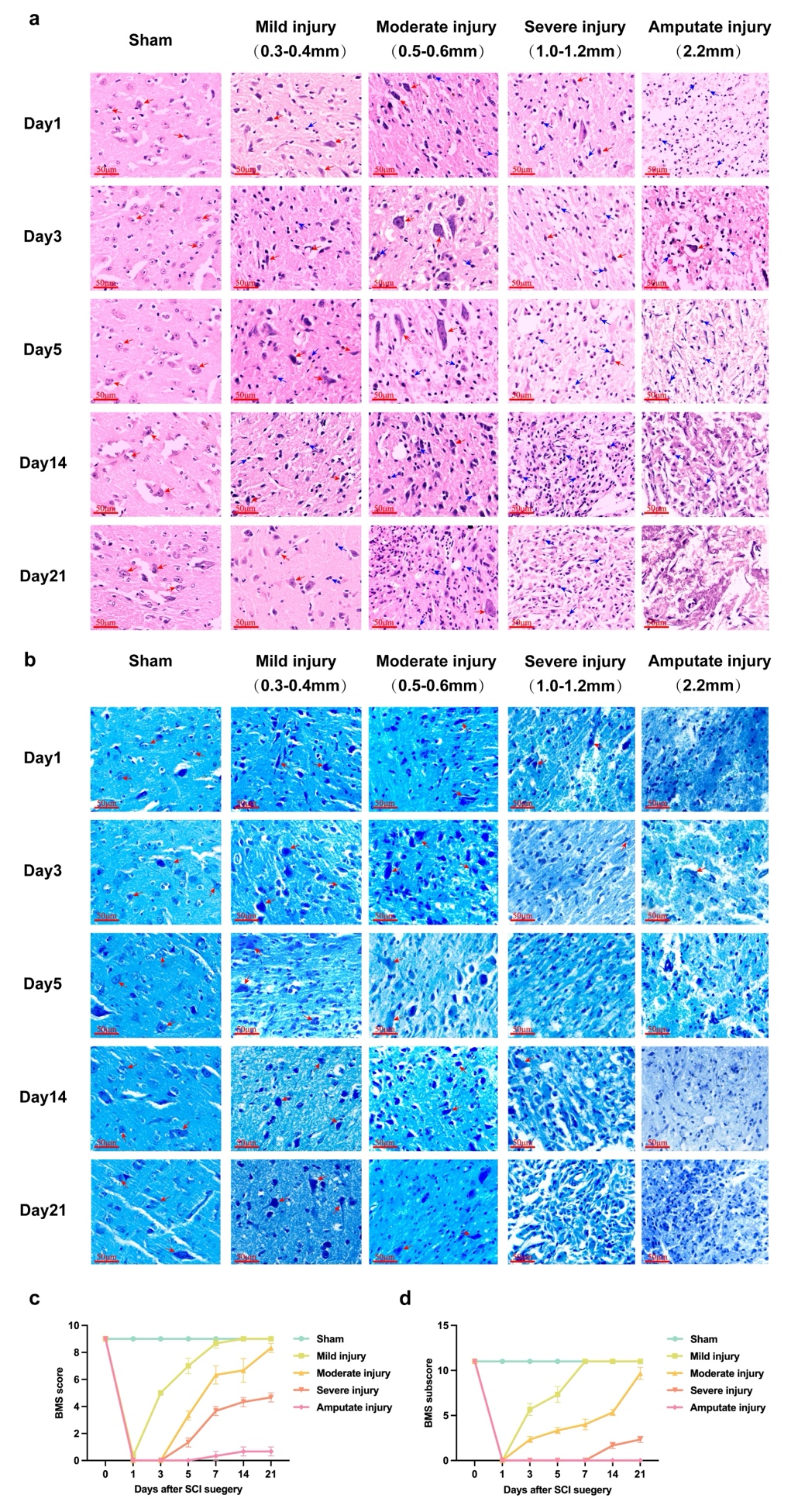


As shown, HE staining was performed to observe pathological changes in the spinal cord, where red arrows indicate neurons and blue arrows indicate inflammatory cells. Each stage of spinal cord injury was observed by looking at the structure of neurons, the infiltration of inflammatory cells and the degree of tissue destruction.

**2. Nissl stain**

**
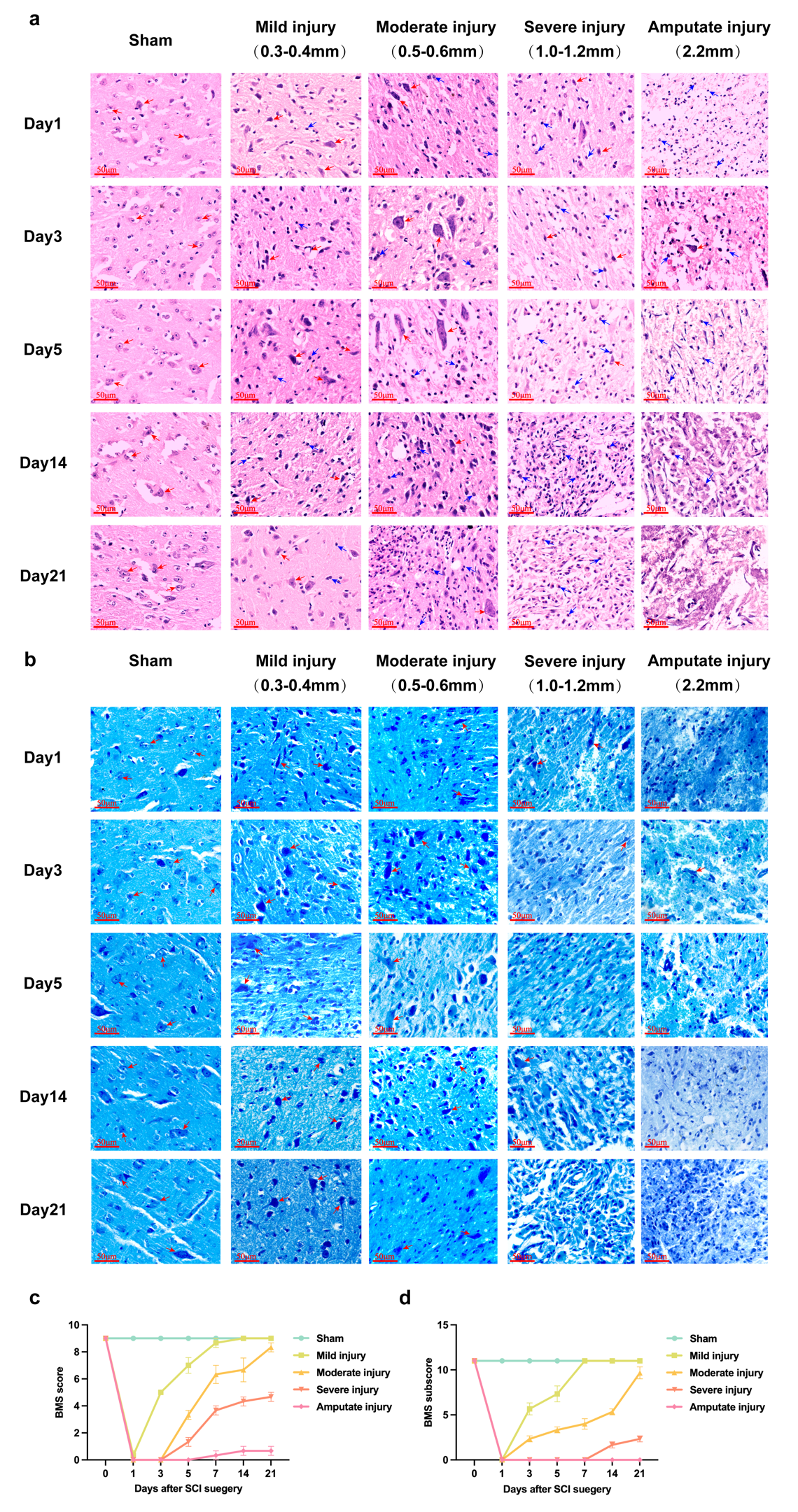
**

**As shown in the figure, the structure of neuronal cells in each group of spinal cord tissue was observed by Niehl's staining, and the structure of neurons could be clearly observed by methylene blue staining, which was used to determine the survival of neurons**
