# Supplementary material for: Expression of cold-inducible RNA-binding protein in mouse spinal cord injury model
Source: PLoS One. 2025 Mar 4;20(3):e0311803. doi: 10.1371/journal.pone.0311803 (PMC11878929; doi:10.1371/journal.pone.0311803)
Supplement: S1 File — (ZIP) [file pone.0311803.s001.zip › Analyze Data/BMS score+BMS subscore.docx]

| BMS score | | | | | | | | | | | | | | | |
| --- | --- | --- | --- | --- | --- | --- | --- | --- | --- | --- | --- | --- | --- | --- | --- |
|  | Sham | | | Mild injury | | | Moderate injury | | | Severe injury | | | Amputate injury | | |
| Day0 | 9 | 9 | 9 | 9 | 9 | 9 | 9 | 9 | 9 | 9 | 9 | 9 | 9 | 9 | 9 |
| Day1 | 9 | 9 | 9 | 1 | 0 | 0 | 0 | 0 | 0 | 0 | 0 | 0 | 0 | 0 | 0 |
| Day3 | 9 | 9 | 9 | 5 | 5 | 5 | 0 | 0 | 0 | 0 | 0 | 0 | 0 | 0 | 0 |
| Day5 | 9 | 9 | 9 | 6 | 8 | 7 | 3 | 4 | 3 | 1 | 1 | 2 | 0 | 0 | 0 |
| Day7 | 9 | 9 | 9 | 9 | 8 | 9 | 5 | 7 | 7 | 4 | 3 | 4 | 0 | 1 | 0 |
| Day14 | 9 | 9 | 9 | 9 | 9 | 9 | 5 | 8 | 7 | 5 | 4 | 4 | 0 | 1 | 1 |
| Day21 | 9 | 9 | 9 | 9 | 9 | 9 | 9 | 8 | 8 | 5 | 4 | 5 | 0 | 1 | 1 |

| BMS subscore | | | | | | | | | | | | | | | |
| --- | --- | --- | --- | --- | --- | --- | --- | --- | --- | --- | --- | --- | --- | --- | --- |
|  | Sham | | | Mild injury | | | Moderate injury | | | Severe injury | | | Amputate injury | | |
| Day0 | 11 | 11 | 11 | 11 | 11 | 11 | 11 | 11 | 11 | 11 | 11 | 11 | 11 | 11 | 11 |
| Day1 | 11 | 11 | 11 | 0 | 0 | 0 | 0 | 0 | 0 | 0 | 0 | 0 | 0 | 0 | 0 |
| Day3 | 11 | 11 | 11 | 5 | 7 | 5 | 2 | 2 | 3 | 0 | 0 | 0 | 0 | 0 | 0 |
| Day5 | 11 | 11 | 11 | 7 | 9 | 6 | 3 | 3 | 4 | 0 | 0 | 0 | 0 | 0 | 0 |
| Day7 | 11 | 11 | 11 | 11 | 11 | 11 | 5 | 3 | 4 | 0 | 0 | 0 | 0 | 0 | 0 |
| Day14 | 11 | 11 | 11 | 11 | 11 | 11 | 6 | 5 | 5 | 2 | 1 | 2 | 0 | 0 | 0 |
| Day21 | 11 | 11 | 11 | 11 | 11 | 11 | 11 | 9 | 9 | 2 | 2 | 3 | 0 | 0 | 0 |
