# Supplementary material for: Expression of cold-inducible RNA-binding protein in mouse spinal cord injury model
Source: PLoS One. 2025 Mar 4;20(3):e0311803. doi: 10.1371/journal.pone.0311803 (PMC11878929; doi:10.1371/journal.pone.0311803)
Supplement: S1 File — (ZIP) [file pone.0311803.s001.zip › Analyze Data/Immunofluorescence analysis.docx]

**1.NeuN**

| Sham | | | Severe injury Day1 | | | Severe injury Day14 | | |
| --- | --- | --- | --- | --- | --- | --- | --- | --- |
| 2.987 | 2.59 | 2.653 | 0.43 | 0.257 | 0.302 | 0 | 0 | 0 |

| Column B | Severe injury Day1 |
| --- | --- |
| vs. | vs. |
| Column A | Sham |
| P value | <0.0001 |
| P value summary | **** |
| Significantly different (P < 0.05)? | Yes |
| One- or two-tailed P value? | Two-tailed |
| t, df | t=18.06, df=4 |
| Mean of column A | 2.743 |
| Mean of column B | 0.3297 |
| Difference between means (B - A) ± SEM | -2.414 ± 0.1336 |
| 95% confidence interval | -2.785 to -2.043 |
| R squared (eta squared) | 0.9879 |

| Column C | Severe injury Day14 |
| --- | --- |
| vs. | vs. |
| Column A | Sham |
| P value | <0.0001 |
| P value summary | **** |
| Significantly different (P < 0.05)? | Yes |
| One- or two-tailed P value? | Two-tailed |
| t, df | t=22.27, df=4 |
| Mean of column A | 2.743 |
| Mean of column C | 0 |
| Difference between means (C - A) ± SEM | -2.743 ± 0.1232 |
| 95% confidence interval | -3.085 to -2.401 |
| R squared (eta squared) | 0.992 |

**2.CIRBP**

| Sham | | | Severe injury Day1 | | | Severe injury Day14 | | |
| --- | --- | --- | --- | --- | --- | --- | --- | --- |
| 0.013 | 0.035 | 0.01 | 0.441 | 0.777 | 0.512 | 1.639 | 3.909 | 2.33 |

| Column B | Severe injury Day1 |
| --- | --- |
| vs. | vs. |
| Column A | Sham |
| P value | 0.0056 |
| P value summary | ** |
| Significantly different (P < 0.05)? | Yes |
| One- or two-tailed P value? | Two-tailed |
| t, df | t=5.435, df=4 |
| Mean of column A | 0.01933 |
| Mean of column B | 0.5767 |
| Difference between means (B - A) ± SEM | 0.5573 ± 0.1025 |
| 95% confidence interval | 0.2726 to 0.8420 |
| R squared (eta squared) | 0.8807 |

| Column C | Severe injury Day14 |
| --- | --- |
| vs. | vs. |
| Column A | Sham |
| P value | 0.0178 |
| P value summary | * |
| Significantly different (P < 0.05)? | Yes |
| One- or two-tailed P value? | Two-tailed |
| t, df | t=3.880, df=4 |
| Mean of column A | 0.01933 |
| Mean of column C | 2.626 |
| Difference between means (C - A) ± SEM | 2.607 ± 0.6718 |
| 95% confidence interval | 0.7413 to 4.472 |
| R squared (eta squared) | 0.7901 |

**3.IBA1**

| Sham | | | Severe injury Day1 | | | Severe injury Day14 | | |
| --- | --- | --- | --- | --- | --- | --- | --- | --- |
| 0 | 0 | 0 | 3.538 | 1.14 | 2.064 | 9.661 | 6.459 | 5.896 |

| Column B | Severe injury Day1 |
| --- | --- |
| vs. | vs. |
| Column A | Sham |
| P value | 0.0323 |
| P value summary | * |
| Significantly different (P < 0.05)? | Yes |
| One- or two-tailed P value? | Two-tailed |
| t, df | t=3.218, df=4 |
| Mean of column A | 0 |
| Mean of column B | 2.247 |
| Difference between means (B - A) ± SEM | 2.247 ± 0.6983 |
| 95% confidence interval | 0.3086 to 4.186 |
| R squared (eta squared) | 0.7214 |

| Column C | Severe injury Day14 |
| --- | --- |
| vs. | vs. |
| Column A | Sham |
| P value | 0.0033 |
| P value summary | ** |
| Significantly different (P < 0.05)? | Yes |
| One- or two-tailed P value? | Two-tailed |
| t, df | t=6.259, df=4 |
| Mean of column A | 0 |
| Mean of column C | 7.339 |
| Difference between means (C - A) ± SEM | 7.339 ± 1.172 |
| 95% confidence interval | 4.083 to 10.59 |
| R squared (eta squared) | 0.9074 |

**3.Tunel**

| Sham | | | Severe injury Day1 | | | Severe injury Day14 | | |
| --- | --- | --- | --- | --- | --- | --- | --- | --- |
| 0 | 0 | 0 | 0.175 | 0.243 | 0.163 | 9.749 | 10.269 | 7.885 |

| Column B | Severe injury Day1 |
| --- | --- |
| vs. | vs. |
| Column A | Sham |
| P value | 0.0015 |
| P value summary | ** |
| Significantly different (P < 0.05)? | Yes |
| One- or two-tailed P value? | Two-tailed |
| t, df | t=7.775, df=4 |
| Mean of column A | 0 |
| Mean of column B | 0.1937 |
| Difference between means (B - A) ± SEM | 0.1937 ± 0.02491 |
| 95% confidence interval | 0.1245 to 0.2628 |
| R squared (eta squared) | 0.9379 |

| Column C | Severe injury Day14 |
| --- | --- |
| vs. | vs. |
| Column A | Sham |
| P value | 0.0002 |
| P value summary | *** |
| Significantly different (P < 0.05)? | Yes |
| One- or two-tailed P value? | Two-tailed |
| t, df | t=12.85, df=4 |
| Mean of column A | 0 |
| Mean of column C | 9.301 |
| Difference between means (C - A) ± SEM | 9.301 ± 0.7237 |
| 95% confidence interval | 7.292 to 11.31 |
| R squared (eta squared) | 0.9764 |
